# Local CpG-*Stat3* siRNA treatment improves antitumor effects of immune checkpoint inhibitors

**DOI:** 10.1016/j.omtn.2024.102357

**Published:** 2024-10-09

**Authors:** Chunyan Zhang, Rui Huang, Lyuzhi Ren, Antons Martincuks, JiEun Song, Marcin Kortylewski, Piotr Swiderski, Stephen J. Forman, Hua Yu

**Affiliations:** 1 Department of Immuno-Oncology, Beckman Research Institute, City of Hope Medical Center, Duarte, CA 91010, USA; 2 DNA/RNA Synthesis Core Facility, Beckman Research Institute, City of Hope Medical Center, Duarte, CA 91010, USA; 3 Department of Hematology & Hematopoietic Cell Transplantation, Beckman Research Institute, City of Hope Medical Center, Duarte, CA 91010, USA

**Keywords:** MT: Oligonucleotides: Therapies and Applications, CpG-*Stat3* siRNA, immunotherapy, immune checkpoint blockade, ICB

## Abstract

Immune checkpoint blockade (ICB) therapy has significantly benefited patients with several types of solid tumors and some lymphomas. However, many of the treated patients do not have a durable clinical response. It has been demonstrated that rescuing exhausted CD8^+^ T cells is required for ICB-mediated antitumor effects. We recently developed an immunostimulatory strategy based on silencing STAT3 while stimulating immune responses by CpG, a ligand for Toll-like receptor 9 (TLR9). The CpG-small interfering RNA (siRNA) conjugates efficiently enter immune cells, silencing STAT3 and activating innate immunity to enhance T cell-mediated antitumor immune responses. In the present study, we demonstrate that blocking STAT3 through locally delivered CpG-*Stat3* siRNA enhances the efficacies of the systemic PD-1 and CTLA4 blockade against mouse A20 B cell lymphoma. In addition, locally delivered CpG-*Stat3* siRNA combined with systemic administration of PD-1 antibody significantly augmented both local and systemic antitumor effects against mouse B16 melanoma tumors, with enhanced tumor-associated T cell activation. Furthermore, locally delivered CpG-*Stat3* siRNA enhanced CD8^+^ T cell tumor infiltration and antitumor activity in a xenograft tumor model. Overall, our studies in both B cell lymphoma and melanoma mouse models demonstrate the potential of combinatory immunotherapy with CpG-*Stat3* siRNA and checkpoint inhibitors as a therapeutic strategy for B cell lymphoma and melanoma.

## Graphical abstract

**Figure:**
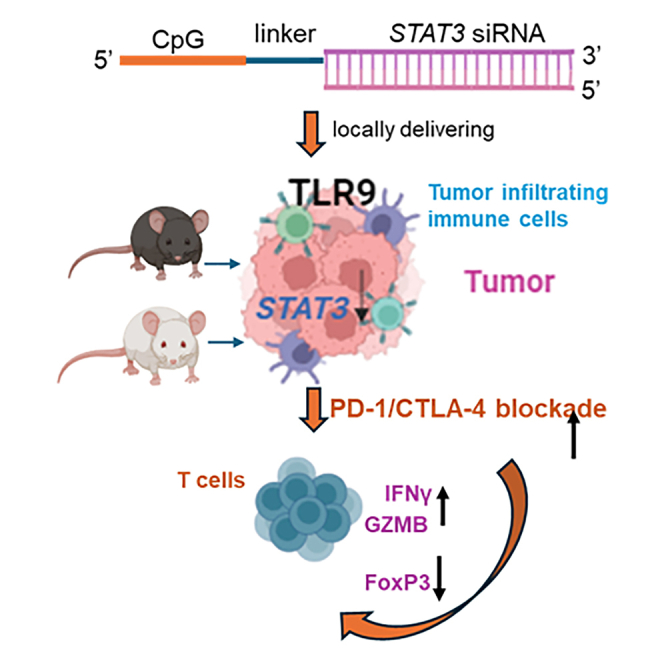

## Teaser

Yu, Zhang, and colleagues demonstrate here that locally delivering CpG-Stat3siRNA enhances the efficacies of systemic PD-1 and/or CTLA4 blockade in two mouse tumor models and a xenograft tumor model. Local injection of CpG-Stat3siRNA also increases PD-1 and/or CTLA4 blockade-induced antitumor CD8^+^ T cell activity.

## Introduction

Therapeutic immune checkpoint blockade (ICB) has profoundly and positively impacted B cell lymphoma and melanoma treatment. However, many treated patients do not respond, and those who have a good initial response may not have durable clinical benefits.^1^^,^^2^^,^^3^ The limited responses to ICB in patients with cancer are largely attributed to a lack of interferon (IFN) signaling and activation of CD8^+^ T cells even after the immune checkpoint is removed.^4^^,^^5^^,^^6^^,^^7^ Extensive studies from our group and others have demonstrated that IFNγ, which is necessary for PD-1-directed therapy responses,^8^^,^^9^ is inhibited in tumor-associated immune cells by STAT3.^10^ STAT3 is also known to promote tumor cell proliferation, survival, and invasion in diverse cancers.^11^^,^^12^^,^^13^^,^^14^^,^^15^^,^^16^ The immunosuppressive role of STAT3 in tumor cells, T cells, B cells, myeloid cells, and dendritic cells (DCs) has been documented extensively.^10^^,^^11^^,^^12^^,^^13^^,^^14^^,^^15^^,^^16^^,^^17^^,^^18^^,^^19^^,^^20^^,^^21^^,^^22^^,^^23^ Additionally, STAT3 signaling in CD4^+^ T cells promotes regulatory T cell (Treg) accumulation in tumors while inhibiting CD8^+^ effector T (T_EFF_)tumor infiltration and antitumor immunity.^12^^,^^18^

STAT3 as a target for inhibiting B cell lymphoma cell survival and activating antitumor immune response has been documented.^24^^,^^25^^,^^26^^,^^27^ We previously developed an immunostimulatory strategy by linking *Stat3* siRNA with CpG, the ligand for Toll-like receptor 9 (TLR9).^20^^,^^25^^,^^28^^,^^29^^,^^30^ CpG not only facilitates the delivery of the small interfering RNA (siRNA) but also, in the absence of STAT3 activity, activates potent antitumor immune responses.^30^ We further showed that both CpG-*Stat3* siRNA and CTLA4 antibody inhibit STAT3 in B malignant cells, leading to tumor cell apoptosis and/or proliferation inhibition.^25^^,^^31^ Additionally, CpG-*Stat3* siRNA activates antitumor T cells by blocking STAT3 in macrophages, B cells, and DCs, while CTLA4 or PD-1 antibody also enables interaction between DCs and T cells to activate antitumor T cell immunity.^20^^,^^32^^,^^33^ Thus, combinatory treatments with CpG-*Stat3* siRNA and CTLA4 or PD-1 antibody may significantly boost the antitumor efficacies of CTLA4 or PD-1 antibody in treating B cell lymphoma and melanoma.

Local treatment can lead to direct antitumor effects at the injected tumor sites and induction of systemic antitumor immune responses.^34^^,^^35^^,^^36^^,^^37^ Both lymph-node-resident B cell lymphoma and cutaneous and subcutaneous melanoma tumors provide the opportunity for local treatment for easier delivery and generally lower toxicity. Previously, we showed that local treatments with CpG-*Stat3* siRNA inhibit both B cell lymphoma^25^ and melanoma tumor growth^20^ while resulting in effective tumor growth inhibition, including complete tumor eradication, when combined with localized radiation therapy.^25^^,^^38^ However, whether CpG-*Stat3* siRNA can boost the efficacies of ICB in these two tumor types remains unknown. The CpG oligonucleotide TLR9 ODN 1826 agonist has been shown to effectively augment the therapeutic potential of checkpoint blockade through locally delivered ODN 1826 and systemic CTLA4 or PD-1 blockade antibody in the B16 melanoma mouse model.^37^ However, high CpG dosing (30 μg/intra-tumorally) was required to enhance the ICB response in this study.^37^ High-dose CpG injections induced sharp and sustained increased local and serum levels of interleukin (IL)-12, IL-6, and tumor necrosis factor alpha (TNF-α), followed by high levels of the acute-phase proteins serum amyloid A^39^ and serum amyloid P (SAP), which could cause strong side effects.^40^^,^^41^ Therefore, we hypothesized that CpG-*Stat3* siRNA, at significantly lower and safer effective CpG concentrations, may enhance the antitumor efficacy of ICB by blocking STAT3 in the tumor-associated myeloid cells, thereby stimulating antigen presentation and activating CD4^+^ and CD8^+^ T cell-mediated antitumor immune responses. Our study shows that at relatively low CpG-*Stat3* siRNA concentrations, local injection can augment the therapeutic potential of PD-1 antibody not only in CpG-*Stat3* siRNA-treated tumors but also in distal non-treated tumors. In the xenograft tumor model, CpG-*Stat3* siRNA local injection can enhance the antitumor effects induced by systemic anti-PD-1 antibody treatment.

## Results

### Local CpG-*STAT3* siRNA treatment enhances anti-lymphoma effects of CTLA4 and PD-1 blockade

**Figure 1:**
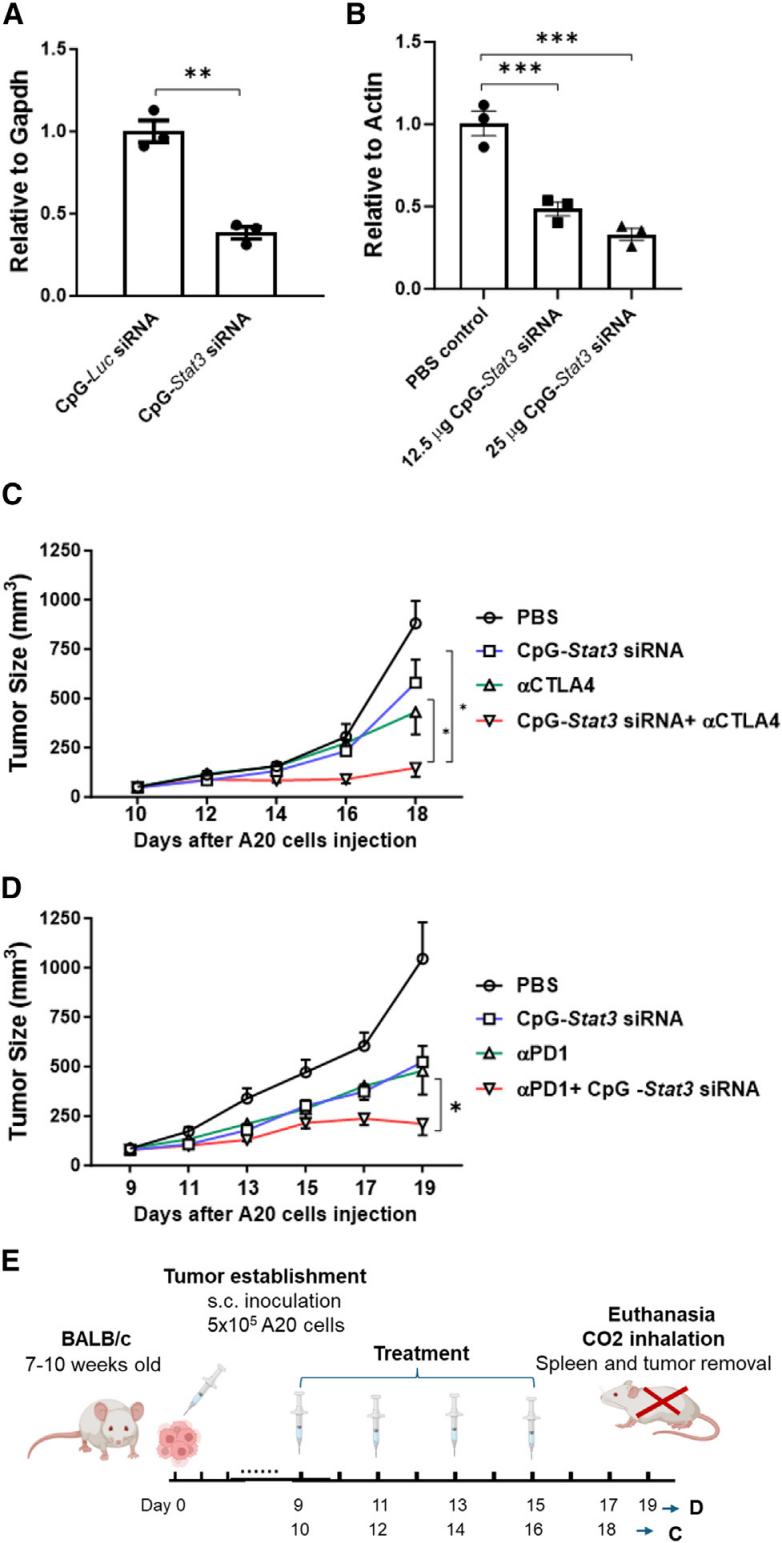
CpG-*Stat3* siRNA gene silencing effect in A20 B cell lymphoma cells and therapeutic effect of CpG-*Stat3* siRNA with or without CTLA4 or PD-1 blockade (A) A20 cells were cultured in the presence of CpG-*Stat3* siRNA or CpG-*Luc*-siRNA for 48 h. CpG-*Stat3* siRNA silences *Stat3* gene expression at the mRNA level, as shown by qPCR. Data are shown as means ± SEM, *n* = 3. (B) BALB/c mice with subcutaneous (s.c) A20 lymphoma tumors were treated by intra-tumoral injections of CpG-*Stat3* siRNA using the indicated amounts of PBS. CpG-*Stat3* siRNA gene silencing effect is shown by qPCR. Data are shown as means ± SEM, *n* = 3 mice. (C and D) BALB/c mice with s.c. A20 lymphoma tumors were treated by intra-tumoral injections of CpG-*Stat3* siRNA, i.p injection of anti-CTLA4 or anti-PD-1 antibodies, or combination treatment with CpG-*Stat3* siRNA and anti-CTLA4 or anti-PD-1 antibodies every other day, starting 9–10 days after tumor implantation (5 × 10^5^ A20 cells/tumor). Data are shown as means ± SEM, *n* = 5. Student’s t test or one-way ANOVA was used for statistical analysis (∗*p* < 0.05, ∗∗*p* < 0.01, and ∗∗∗*p* < 0.001). Tumor size was monitored every other day. (E) *In vivo* study design for (C) and (D).

Although some advances have been made in treating patients with cancer with ICB, the efficacy is limited, which is contributed to by the lack of CD8^+^ T cell activation.^2^^,^^6^ We have shown previously that CpG-*Stat3* siRNA treatment *in vivo* silences *Stat3* in DCs, B cells, and macrophages, leading to T cell activation and potent antitumor immunity.^20^ We further showed that both CpG-*Stat3* siRNA and CTLA4 antibody inhibit STAT3 in B malignant cells, leading to tumor cell apoptosis and/or growth inhibition.^25^^,^^31^ Here, we assessed whether silencing *Stat3* with CpG-*Stat3* siRNA can significantly enhance the efficacies of CTLA4 antibody therapy in B cell lymphoma by augmenting the immunostimulatory effects. For our experiments, we selected the commonly used mouse A20 B cell lymphoma model, which exhibits TLR9 expression and constitutive Stat3 activation.^25^ We have previously shown that CpG-*Stat3* siRNA efficiently targeted A20 tumor cells to silence *Stat3*.^20^^,^^25^ To evaluate gene silencing effect of CpG-*Stat3* siRNA, we used quantitative real-time PCR (real-time qPCR) analysis of *Stat3* mRNA of CpG-*Stat3* siRNA-treated A20 tumor cells. The effect of *Stat3* gene silencing was observed in cultured A20 cells at 48 h since the start of CpG-*Stat3* siRNA treatment (Figure 1A). Next, we tested the feasibility of using this strategy for targeting Stat3 *in vivo*. BALB/c mice with established, subcutaneously engrafted (s.c.-engrafted) A20 lymphoma tumors were treated with PBS or CpG-*Stat3* siRNA at 12.5 or 25 μg/mouse by daily intra-tumoral (IT) injections. One day after the third injection of PBS or CpG-*Stat3* siRNA, mice were euthanized, and the tumor tissues were harvested to assess *Stat3* mRNA expression by real-time qPCR. The CpG-*Stat3* siRNA treatment reduced the *Stat3* mRNA expression in a dose-dependent manner (Figure 1B).

We next tested whether combining CpG-*Stat3* siRNA with CTLA4 or PD-1 inhibition would lead to superior antitumor effects relative to the single agents in the mouse B cell lymphoma. As shown in Figures 1C and 1D, silencing *Stat3* through locally delivered CpG-*Stat3* siRNA significantly enhanced the antitumor efficacies of systemic administration of PD-1- and CTLA4-specific antibodies in mice bearing the A20 B cell lymphoma arresting tumor growth. However, locally delivered CpG*-scramble siRNA* did not significantly increase the antitumor effects of PD-1 or CTLA4 antibody (Figure S1).

### Combining CpG-*Stat3* siRNA with CTLA4 or PD-1 blockade induces significantly higher activities of tumor-infiltrating T cells

**Figure 2:**
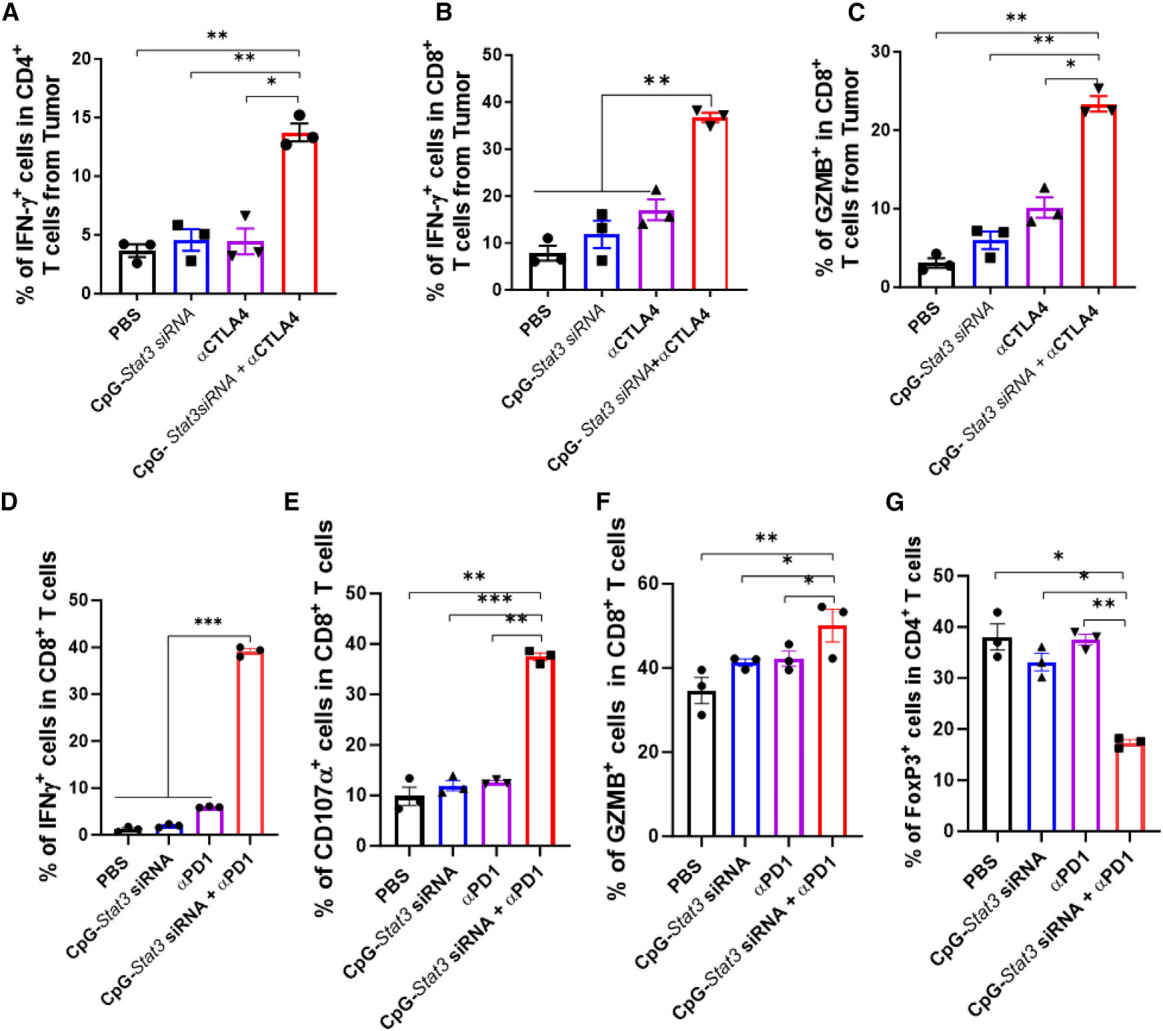
Local CpG-*Stat3* siRNA treatment significantly enhances the antitumor effector functions of tumor-infiltrating T cells in mice treated with CTLA4 or PD-1 blockade Single-cell suspensions prepared from A20 tumors from mice receiving indicated treatments were analyzed by flow cytometry for IFNγ (A, B, and D), CD107α (E), granzyme B (C and F), and FoxP3 (G) expression in T cells. Flow cytometry data showing IFNγ^+^ or GzmB^+^ cell frequencies in tumor-infiltrating CD4^+^ or CD8^+^ T cells. Data are shown as means ± SEM (*n* = 3, *n* is for number of samples, each of which was pooled from 2–3 mice), One-way ANOVA was used for statistical analysis (∗*p* < 0.05, ∗∗*p* < 0.01, and ∗∗∗*p* < 0.001).

Tumor infiltration of activated cytotoxic CD8^+^ T cells is critical for the successful outcome of ICB therapy.^2^^,^^6^^,^^42^ Therefore, we determined whether T cell activation is a critical contributor for the antitumor effect of combinatory immunotherapy with CpG-*Stat3* siRNA and checkpoint blockades. Combinatory treatment with CpG-*Stat3* siRNA and CTLA4 antibody in A20 lymphoma tumor-bearing mice significantly increased the percentages of IFNγ and/or granzyme B (GZMB) producing CD4^+^ T cells and CD8^+^ T cells in tumors compared to either CpG-*Stat3* siRNA or CTLA4 antibody alone (Figures 2A–2C). Similarly, combinatory treatments with CpG-*Stat3* siRNA and PD-1 antibody in A20 lymphoma tumor-bearing mice led to significantly higher percentages of activated CD8^+^ T cells positive for IFNγ, CD107α, which measures T cell cytotoxicity, and GZMB at the tumor sites (Figures 2D–2F). Furthermore, combinatory treatment led to the inhibition of FoxP3^+^ Tregs (Figure 2G). These results support that local CpG-*Stat3* siRNA treatment enhances antitumor effects of CTLA4 or PD-1 blockade by increasing the antitumor effector functions of tumor-infiltrating T cells.

### Local CpG-*STAT3* siRNA combined with PD-1 antibody results in systemic antitumor effects against melanoma in mice

**Figure 3:**
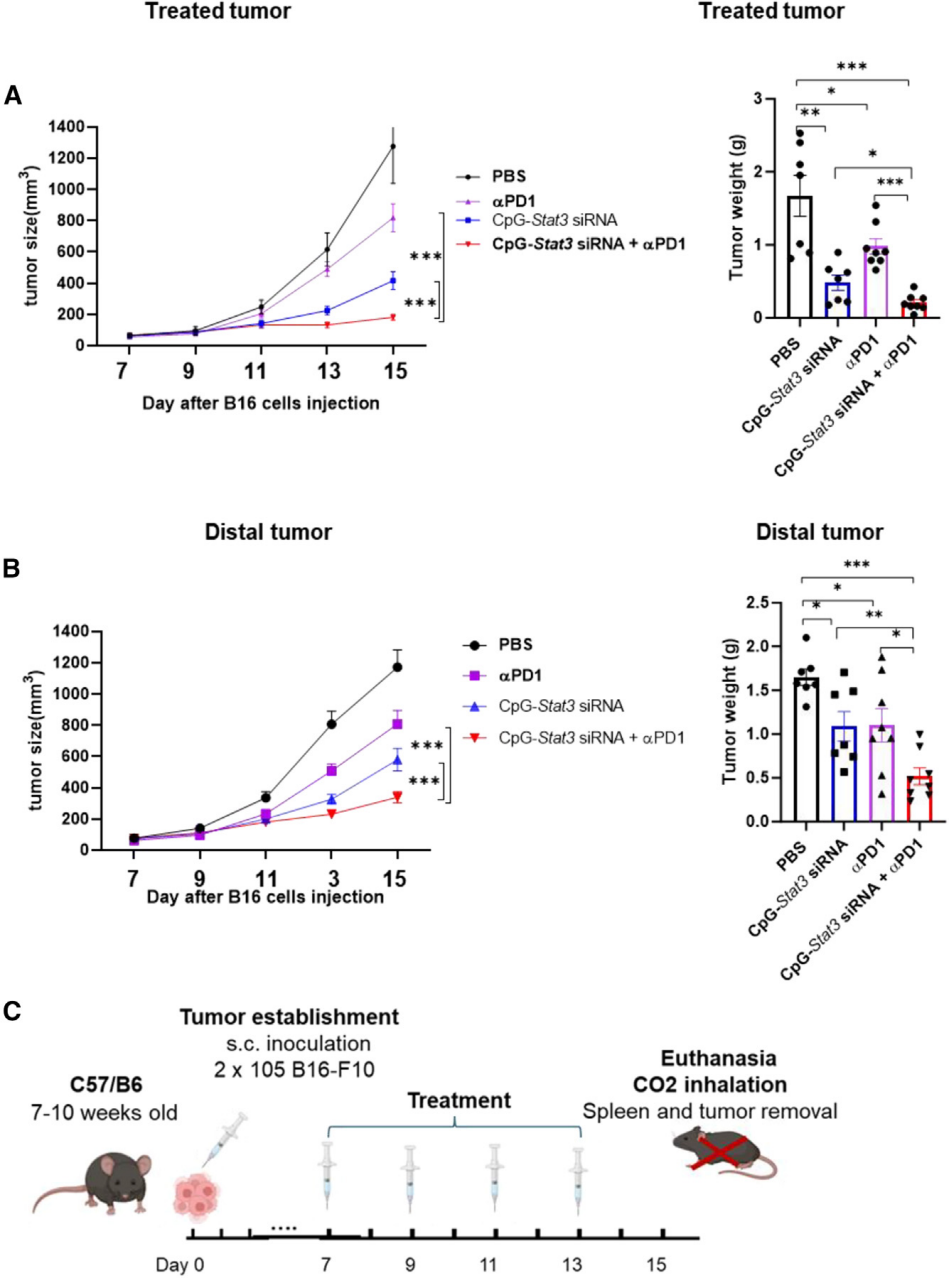
Combination treatment with intra-tumoral CpG-*Stat3* siRNA and systemic PD-1 antibody reduces growth of both treated and distal tumors C57BL/6 mice were injected with 2 × 10^5^ B16 melanoma cells on both right and left flanks. The left flank tumors were treated by intra-tumoral injections of CpG-*Stat3* siRNA, i.p injection of anti-PD-1 antibody, or CpG-*Stat3* siRNA and anti-PD-1 every other day, starting day 7 post-tumor challenge (2 × 10^5^ B16-F10 cells/tumor), *n* = 7–8. Both treated tumor (A) and distal tumor (B) size was monitored every other day. Data are shown as means ± SEM, Student’s t test and one-way ANOVA were used for statistical analysis (∗*p* < 0.05, ∗∗*p* < 0.01, and ∗∗∗*p* < 0.001). (C) *In vivo* study design for (A) and (B).

We previously showed that local treatments with CpG-*Stat3* siRNA inhibit melanoma tumor growth by silencing *Stat3* and activating tumor-infiltrating immune cells.^20^ However, the ideal cancer therapy should not only inhibit local tumor regression but also induce a systemic antitumor immunity that could effectively eradicate distant metastases. Whether CpG-*Stat3* siRNA can enhance both local and systemic antitumor responses of ICB in melanoma remains unknown. Therefore, we evaluated whether combinatory treatment with locally delivered CpG-*Stat3* siRNA and systemic PD-1 antibody treatment could lead to both local and systemic antitumor effects. We used a bilateral tumor model in which the C57BL/6 mice were challenged at both flanks by mouse B16 melanoma tumor cells through s.c. injection. Only one tumor was treated with locally delivered CpG-*Stat3* siRNA to assess local and systemic effects of single and combinatory treatments. Our results show that local CpG-*Stat3* siRNA treatment suppressed the growth of both treated and non-treated distal tumors (Figure 3), suggesting the generation of systemic antitumor immune responses. In addition, combination treatment with CpG-*Stat3* siRNA and systemic PD-1 antibody significantly enhanced the systemic antitumor responses compared to either CpG-*Stat3* siRNA or PD-1 antibody treatment alone (Figure 3). These findings provide evidence that CpG-*Stat3* siRNA local treatment can enhance the PD-1 antibody-mediated systemic antitumor response.

### Combination of the local CpG-*Stat3* siRNA treatment with PD-1 blockade promotes tumor T cell recruitment and activity

**Figure 4:**
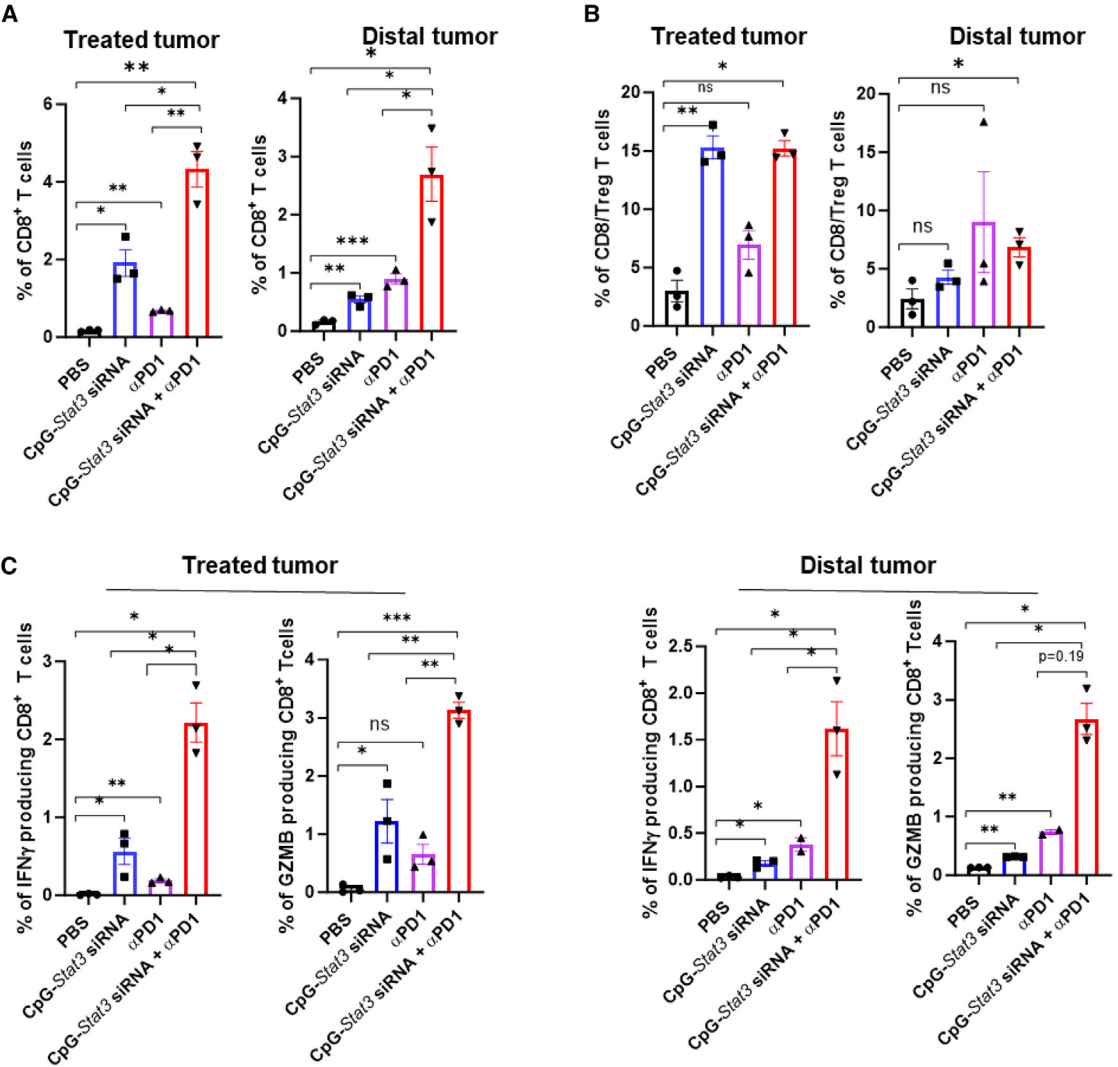
Intra-tumoral CpG-*Stat3* siRNA and systemic PD-1 antibody combined treatment significantly increases CD8^+^ T cell tumor infiltration and activity in treated and distal tumors Single-cell suspensions from the *B16* tumors from mice with indicated treatments were analyzed by flow cytometry for CD8^+^ (A) and CD8^+^/CD4^+^FoxP3^+^ immune cells (B) as well as IFNγ^+^ or GzmB^+^ CD8^+^ (C) cells from the indicated treatments. Data were shown as means ± SEM, *n* = 3 (*n* is for number of samples, each of which was pooled from 2–3 mice). One-way ANOVA was used for statistical analysis (∗*p* < 0.05, ∗∗*p* < 0.01, and ∗∗∗*p* < 0.001).

To assess the effect of CpG-*Stat3* siRNA/anti-PD1 combination treatment on T cell tumor infiltration and activation, we performed experiments using bilateral B16 tumors as described above. On day 15 after tumor implantation, we harvested the tumor tissues and prepared single-cell suspension, followed by antibody staining and flow cytometric analysis. Compared to the PBS control, local CpG-*Stat3* siRNA treatment enhanced the tumor infiltration of CD8^+^ T cells (Figure 4A). IT ratios of CD8^+^ T cells vs. Tregs in treated tumors were significantly increased (Figure 4B). Consistent with the antitumor effects of each treatment in Figure 3, either local CpG-*Stat3* siRNA or systemic PD-1 antibody treatment improved CD8^+^ T cell activity with increased IFNγ and GZMB producing CD8^+^ T cell frequency, but the most significant enhancement of the activated CD8^+^ T cells in bilateral tumors was observed in mice given the combinatory treatment (Figure 4C). These results indicated that the combination treatment with local CpG-*Stat3* siRNA and systemic PD-1 antibody increases the tumor infiltration of activated T cells.

### Local CpG-*Stat3* siRNA treatment systematically increases IFNγ and GZMB production

In addition to immune cells, cytokines also play a critical role in antitumor immune therapy. Both IFNγ and GZMB are well known for their direct cytotoxic effects on tumor cells and being able to stimulate production of immune-stimulating cytokines.^43^^,^^44^^,^^45^ Thus, we co-cultured B16 tumor cells with splenic cells from B16 tumor-bearing mice receiving various treatments. 3 or 4 days after co-culturing, the levels of IFNγ and GZMB in the cultured cell supernatants were measured by ELISA. Both IFNγ and GZMB were noticeably increased, particularly in the combinatory treatment group at day 4 after co-culturing, although there was also a significant induction of IFNγ and GZMB in the local CpG-Stat3 siRNA treatment group (Figure S2). These findings suggest that local CpG-Stat3 siRNA treatment induces and enhances systemic IFNγ and GZMB production triggered by PD-1 antibody treatment.

### Local CpG-*Stat3* siRNA treatment enhances PD-1 blockade-induced antitumor effects in a melanoma xenograft tumor model

**Figure 5:**
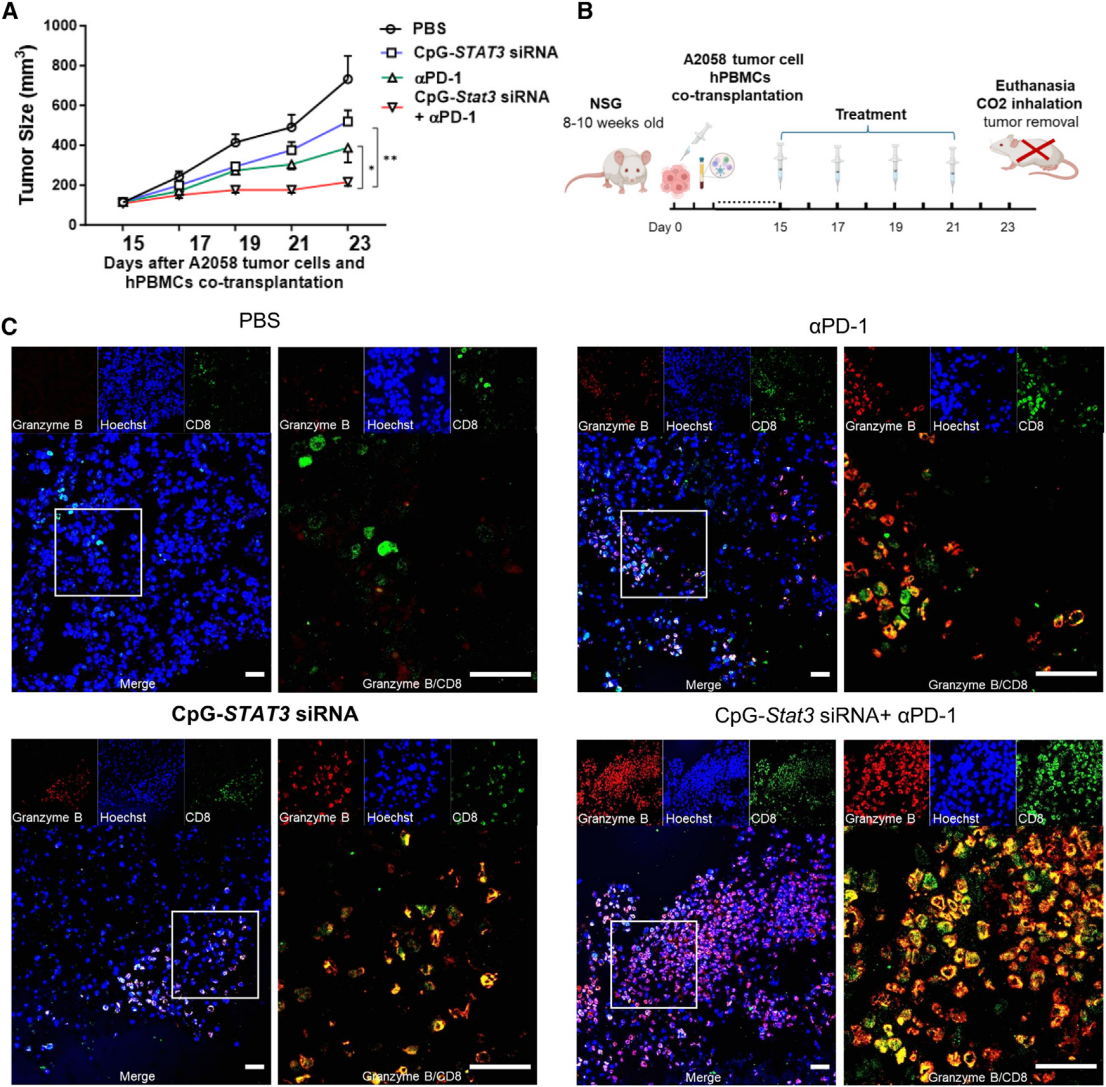
Local CpG-*Stat3* siRNA treatment enhances PD-1 blockade-induced antitumor effects in a melanoma xenograft tumor model (A) Freshly isolated hPBMCs were mixed with A2058 tumor cells at 1:4 (hPBMCs:A2058 cells) and implanted subcutaneously in the left flank of NSG mice. The mice were treated by intra-tumoral injections of CpG-*Stat3* siRNA or i.p injections of anti-PD-1 antibody, alone or in combination, every other day, starting day 15 post-tumor challenge (5 × 10^6^ A2058 tumor cells and 1.25 × 10^6^ hPBMCs/tumor), *n* = 8. Tumor size was monitored. Data are shown with means ± SEM, Student’s t test was used for statistical analysis (∗*p* < 0.05, ∗∗*p* < 0.01, and ∗∗∗*p* < 0.001). (B) *In vivo* study design for (A). (C) Representative immunofluorescence images of CD8 (green), granzyme B (red), and Hoechst (blue) on the tumor tissue sections as indicated. Scale bars represent 50 μm.

NSG mice were implanted with a mixture of human peripheral blood mononuclear cells (hPBMCs) and human melanoma A2058 cells, followed by local CpG-*Stat3* siRNA injection or systemic PD-1 antibody treatment, alone or in combination. Compared to either local CpG-*Stat3* siRNA or systemic PD-1 antibody treatment alone, the combinatory treatment resulted in significantly more tumor inhibition (Figure 5A). Moreover, decreased tumor cell proliferation was observed in the mice given the combinatory treatment, as indicated by reduced Ki67^+^ cells in HMB-45^+^ tumor areas (HMB45 is a human melanoma marker) (Figure S3). This is accompanied by significantly increased infiltration of GZMB^+^ CD8^+^ T cells in the tumors in mice treated with the local CpG-*Stat3* siRNA and systemic PD-1 antibody combination (Figure 5C). In addition, CpG-*Stat3* siRNA local treatment induced p-STAT3 downregulation in CD11b^+^ myeloid cells (Figure S4). These findings further suggest that CpG-*Stat3* siRNA local treatment can enhance the PD-1 antibody-mediated systemic antitumor response in a human melanoma xenograft model.

## Discussion

Despite the promising outcomes of anti-CTLA4 and anti-PD-1 antibodies in treating cancer, including solid tumors and hematopoietic malignancies,^1^^,^^3^^,^^6^^,^^46^ many of the treated patients do not respond well to the ICB, and those who initially do may not have durable clinical benefits.^1^^,^^2^^,^^3^ Thus, there is an urgent need to develop immunotherapeutic strategies to improve the efficacy of ICB. The limited responses to ICB in patients with cancer, including those with B cell lymphoma and melanoma, are largely attributed to the lack of IFNγ production required for the activation of CD8^+^ T cells.^4^^,^^5^^,^^6^^,^^7^ Extensive studies from our group have demonstrated that STAT3, which is persistently activated in multiple types of immune cells in the tumor microenvironment, contributes to the suppression of IFNγ production in the tumor microenvironment.^10^^,^^20^^,^^23^

In this study, we evaluated the antitumor effects of local CpG*-Stat3* siRNA treatment at tumor sites to boost the antitumor effects of CTLA4 and/or PD-1 antibody systemic treatments in the A20 mouse lymphoma model and the B16 melanoma model. Using primary tumors as the injection site has been well documented.^36^^,^^47^^,^^48^ This approach has several merits, including easier delivery, reduced drug dosing and associated toxicity, and having the potential to stimulate systemic antitumor immune responses. Our results demonstrate that combining CpG*-Stat3* siRNA with CTLA4 or PD-1 antibody suppressed A20 lymphoma tumor growth more effectively than either CpG*-Stat3* siRNA or CTLA4 or PD-1 antibody alone. In the case of the B16 melanoma tumor model, we showed that CpG*-Stat3* siRNA local treatment significantly potentiates the antitumor effects of PD-1 antibody, not only at the tumors that have received the treatment but also at non-treated distal tumors. The antitumor effects on the distal non-treated tumors provide evidence supporting the activation of systemic antitumor immune responses. The detection of increased tumor CD8^+^ T cell infiltrates that are activated and able to produce IFNγ and GZMB and reduced tumor-associated Tregs when CpG*-Stat3* siRNA is added to ICB therapy show that CpG*-Stat3* siRNA can serve as an effective adjuvant for ICB. The finding that CpG*-Stat3* siRNA can elevate IFNγ and GZMB production by splenic cells in mice receiving anti-PD-1 treatment further supports that CpG*-Stat3* siRNA can boost the antitumor efficacy of anti-PD-1 treatment. We have noted that the antitumor effectiveness is stronger at the treated tumor than the distal tumor. Similar findings were reported,^49^^,^^50^ likely due to concentrated immunostimulatory factors produced at the tumor site receiving CpG-Stat3siRNA, attracting more antigen-presenting cells and other immune cells, including both innate immune cells and T cells.^20^^,^^49^^,^^50^

Pioneering studies by others have demonstrated CpG as an immunostimulatory molecule that activates innate immunity and induces systemic immune response through TLR9 in solid and blood cancer, including B cell lymphoma and melanoma.^37^^,^^48^^,^^50^^,^^51^^,^^52^ However, high CpG dosing (30–100 μg/intra-tumorally) is required to enhance the antitumor immune response in these studies.^37^^,^^48^^,^^50^^,^^51^ It has been demonstrated that high-dose CpG injections can cause strong side effects associated with innate immune cell activation or toxicities related to chemical modification of the oligonucleotide, such as phosphorothioation.^40^^,^^41^^,^^52^ CpG in CpG*-Stat3* siRNA acts as not only an immune-stimulatory molecule but also a carrier for *Stat3* siRNA. In the present study, we showed that a local injection of relatively low-dose (0.5–2.5 mg/kg body weight) CpG-*Stat3* siRNA, in which CpG represents roughly a quarter of the conjugate, is effective in increasing both CTLA4 and PD-1 antibody-mediated antitumor immune responses, including recruitment and activation of tumor CD8^+^ T cells, reduction of tumor-infiltrating CD4^+^ Tregs, and improved antitumor effects. *Stat3* gene silencing by *Stat3* siRNA in the CpG*-Stat3* siRNA conjugate has been shown to reduce the expression of immunosuppressive factors while increasing expression of immunostimulatory molecules including IFNγ.^20^^,^^29^ Because of this, it has been shown that CpG*-Stat3* siRNA is more effective in activating antitumor immune responses than CpG stimulation alone.^20^ In addition to CpG, immune checkpoint antibodies are associated with autoimmune toxicities.^53^ Recently, similar therapies in cancer treatment have been reported.^54^^,^^55^ One study showed that RNAi-mediated silencing of STAT3 and PD-L1 led to tumor growth inhibition by activating tumor-infiltrating immune cells, while RNAi-mediated silencing of STAT3/PD-L1 in tumor-associated immune cells induces robust antitumor effects in immunotherapy resistant tumors.^54^ Another publication shows that silencing STAT3 via peptidomimetic lipid nanoparticles (LNP)-mediated systemic delivery of RNAi downregulated PD-L1, resulting in the inhibition of melanoma growth.^55^ Although there are some similarities to these reports, our current study shows that local injection of CpG-*Stat3* siRNA can boost the effects PD-1 antibody systemic treatment at reduced doses (100 vs. 200 μg per mouse, which is expected to reduce toxicity) to induce systemic antitumor immune responses.

Tumor cells from melanoma and many types of B cell lymphomas require persistently activated STAT3 for growth and/or survival.^25^^,^^26^^,^^56^^,^^57^ Our previous studies have shown that TLR9 is required to process CpG*-Stat3* siRNA to be an effective siRNA.^58^ B cell lymphoma cells, including mouse A20 tumor cells, express TLR9.^25^^,^^26^ Follow-up studies are needed to demonstrate the contribution of *Stat3* silencing in the tumor cells in enhancing the efficacies of ICB in A20 tumor model. Several tumor cells, especially cancer stem cells, in some solid tumors, including glioma, express TLR9.^28^ The glioma cancer stem cells can also process CpG*-Stat3* siRNA into effective siRNA.^28^ Nevertheless, the B16 melanoma cells do not display TLR9.^20^ Therefore, at least in the B16 tumor model, CpG*-Stat3* siRNA-induced enhancement of the antitumor effects of PD-1 is largely contributed by immune activation.

Taken together, the results of our study showed that local administration of low-dose CpG-*Stat3* siRNA can enhance the therapeutic potential of IBC in B cell lymphoma and melanoma mouse tumor models, supporting further testing and development of CpG-*Stat3* siRNA and ICB combinatory treatment for clinical application.

## Materials and methods

### Cell lines

Mouse lymphoma cell line A20 (American Type Culture Collection [ATCC]; A-20) was cultured in RPMI containing 10% fetal bovine serum (FBS; Omega Scientific) and 1× antibiotic antimycotic (AA; Gibco), supplemented with 0.05 mM 2-mercaptoethanol (2-ME; Gibco). Either mouse melanoma cell line B16 (ATCC) or human melanoma cell line A2058 (ATCC) was cultured in Dulbecco’s modified Eagle’s medium (DMEM) containing 10% FBS and 1× AA.

### *In vivo* mouse experiments

Mouse care and experimental procedures were performed under pathogen-free conditions in accordance with established institutional guidance and approved protocols (IACUC: 08026) from the Institutional Animal Care and Use Committee at the Beckman Research Institute of the City of Hope Medical Center. C57BL/6 and BALB/c mice were obtained from Jackson Laboratory. 8- to 10-week-old female NSG mice from The Jackson Laboratory or animal resource core facility were used to establish the human melanoma xenograft model. For the A20 lymphoma mouse model, we injected 5 × 10^5^ A20 tumor cells s.c. into BALB/c mice. When the tumors reached an average diameter of approximately 6–7 mm (9–10 days after A20 tumor cell injection), mice with similar average tumor sizes were randomly divided into four groups. Then, we treated A20 tumor-bearing mice with IT injections of CpG-*Stat3* siRNA (0.5 mg/kg body weight), intraperitoneal (i.p.) injections of CTLA4/PD-1 antibody (100 μg/mouse), or combination treatment with CpG-*Stat3* siRNA and CTLA4 (clone 9D9, BioXcell)/PD-1(clone 29F.1A12 BioXcell) antibody every other day. For the IT injection, we used a fine (27G) needle to deliver a minimal volume of 50 μL per tumor. Tumor growth was monitored every other day with caliper measurement. For the B16 melanoma mouse model, C57BL/6 mice were s.c. injected with 2 × 10^5^ B16 tumor cells on the right and left flanks. The left flank tumors were treated by IT injections of CpG-*STAT3* siRNA (0.5 mg/kg body weight), i.p injection of PD-1 antibody (100 μg/mouse), or combination treatment with CpG-*STAT3* siRNA and αPD1 every other day, starting 7 days after challenge with 2 × 10^5^ B16 cells. Tumor growth was monitored as described above. For the human melanoma xenograft model, freshly isolated hPBMCs were mixed with cultured A2058 tumor cells at 1:4 (hPBMCs:A2058 cells) and implanted s.c. into the left flank of NSG mice. The tumors were treated by IT injections of CpG-*Stat3* siRNA (2.5 mg/kg, body weight), i.p injection of anti-PD-1 antibody (100 μg/mouse), or CpG-*Stat3* siRNA and anti-PD-1 every other day, starting day 15 post-tumor challenge (1.25 × 10^6^ hPBMCs and 5 × 10^6^ A2058 tumor cells). Tumor size was monitored every other day.

### Oligonucleotide design and synthesis

The sequences of mouse cell-specific CpG1668(B)-siRNAs and the chemical modification have been described previously.^20^ The sequences of single-stranded constructs are listed below.

Mouse Stat3 siRNA (SS): 5′-CAGGGUGUCAGAUCACAUGGGCUAA-3′.

CpG1668-mouse Stat3 siRNA (AS): 5′-TCCATGACGTTCCTGATGCT-linker-UUAGCCCAUGUGAUCUGACACCCUGAA-3′.

Human Stat3 siRNA (SS): 5′-GGAAGCUGCAGAAAGAUACGACUGA-3′.

CpG(A)-STAT3 siRNA (AS): 5′-G∗G∗TGCATCGATGCAGG∗G∗G∗G∗G-linker-UCAGUCGUAUCUUUCUGCAGCUUCCGU-3′.

### Real-time qPCR

Total RNAs from various cell populations were purified with the RNeasy system according to the manufacturer’s instructions (QIAGEN). RNA (0.5–1 μg) was reverse transcribed to cDNA using the iScript cDNA Synthesis Kit (Bio-Rad), and real-time PCR reactions were performed as described previously.^10^ Specific primers for mouse Stat3 (Qiagen, PPM04643F), Actin (Qiagen, PPM02945B), and Gapdh (Qiagen, PPM02946E) were purchased from SA Bioscience and Qiagen. Each primer set was validated using a standard curve across the dynamic range of interest with a single melting peak. Samples were run in triplicate and expressed as means ± standard error of the mean (SEM).

### hPBMC preparation

The use of anonymous discarded blood samples was approved by the City of Hope Institutional Review Board and exempt from the informed consent requirement. PBMCs were isolated from human blood by density gradient centrifugation using Ficoll-Paque Plus (GE Healthcare Biosciences).

### Immunofluorescent staining and confocal microscopy

Mouse tumor tissue sections were obtained from melanoma tumor-bearing NSG mice and subsequently embedded in optimal cutting temperature (OCT) block, stained for immunofluorescence with specific primary and fluorophore-conjugated secondary antibodies, and imaged by confocal microscopy as previously described.^10^ Briefly, the tissue sections from the OCT-frozen tumor tissues were fixed in 4% paraformaldehyde, permeabilized with methanol, and blocked in PBS containing 5% goat serum. Samples were stained overnight at 4°C with the following primary antibodies: HMB45 (ab787, Abcam), Ki67 (VP-RM04, Vector), CD11b (clone LM2/1.6.11, Santa Cruz Biotechnology), CD8 (clone SP16, Thermo Scientific), GZMB (Cell Signaling Technology), and p-STAT3 (clone D3A7, Cell Signaling Technology). The next day, the slides were incubated with secondary antibodies for 2 h (Alexa Fluor 488 goat anti-rabbit, Alexa Fluor 555 goat anti-mouse, Thermo Scientific). Afterward, slides were mounted, and confocal imaging was performed with a Zeiss LSM 880 confocal microscope. Staining quantification was performed by ZEN 2.3 lite software and plotted in GraphPad Prism 9. For Ki67 analysis, the nuclear positive percentage within the tumor area was calculated using the software QuPath-0.4.2.

### Intracellular staining and flow cytometry

#### Antibodies

Fluorochrome-conjugated monoclonal antibodies against CD45 (clone I3/2.3), CD4 (clone GK1.5), and CD8 (clone 53-5.8) were purchased from BioLegend (San Diego, CA, USA). Antibodies against FoxP3 (clone FJK-16s), CD107α (clone 1D4B), GZMB (clone OA16A02), and IFNγ (clone XMG1.2) were obtained from eBioscience and BioLegend.

#### Single-cell suspension preparation

To prepare single-cell suspensions, tumor tissue was dissected into approximately 1–5 mm^3^ fragments and digested with collagenase type D (2 mg/mL; Roche) and DNase I (1 mg/mL; Roche) for 30–45 min at 37°C. Digests were filtered through 70 μm cell strainers and centrifuged at 1,500 rpm for 5 min. Single-cell suspensions from spleens were prepared as mentioned above. After red blood cell lysis (Sigma-Aldrich), single-cell suspensions were filtered, washed, and resuspended in fluorescence-activated cell sorting (FACS) buffer (2% FBS in Hank’s balanced salt solution without Ca^+^, Mg^+^, or phenol red).

Intracellular staining and flow cytometric analysis were performed as previously described.^10^ Single-cell suspensions (some of which were pooled from tumors harvested from 2–3 mice) were stimulated for 5 h with PMA (5 ng/mL, Sigma) and ionomycin (500 ng/mL, Sigma) in the presence of a protein transport inhibitor (monensin 1,000×, BioLegend). Cells were blocked with anti-CD16/CD32 antibody (TruStain FcX PLUS anti-mouse CD16/32, BioLegend) and incubated for 15 min on ice with PECy7-, Alexa Fluor 700-, Pacific Blue- (or v450), and APC-Cy7 (or Alexa Fluor-e780)-conjugated antibodies (1:100, CD4, CD8) purchased from BioLegend. After cell surface marker staining, cells were fixed and permeabilized using the BD Cytofix/Cytoperm Fixation/Permeabilization Solution Kit (BD) for IFNγ and GZMB staining or eBioscience Foxp3/Transcription Factor Staining Buffer Set (eBioscience) for FoxP3 staining. Cells were then incubated with FITC and PE-conjugated antibodies (1:100, IFNr or FoxP3, GZMB) purchased from BioLegend. Aqua LIVE/DEAD, used for cell viability, was purchased from Invitrogen. Cells were washed twice before analysis on the BD LSR Fortessa flow cytometer (Beckman Coulter Genomics).

### Cytokine measurement

Splenic cells (1 × 10^5^) from B16 tumor-bearing mice were co-cultured with B16 tumor cells (2 × 10^4^) in a well of the 96-well plate for 3 or 4 days. Culture medium was collected to determine IFNγ and GZMB levels by ELISA (R&D).

### Statistical analysis

Statistical analyses were performed using GraphPad Prism 7 software. Statistical comparisons between groups were performed using the unpaired Student’s t test to calculate two-tailed *p* values. Statistical significance values were set as ∗*p* < 0.05, ∗∗*p* < 0.01, and ∗∗∗*p* < 0.001. Multiple group comparisons were conducted using one-way ANOVA. A *p* value less than 0.05 would be considered statistically significant, and ns means not significant. Data are presented as mean ± SEM. *p* value and *n* can be found in the main and supplemental figure legends.

## Data and code availability

All reagents and data generated from this study are available from the corresponding author upon reasonable request.

## Acknowledgments

We thank the staff members at the animal facilities at City of Hope for their dedication. We also acknowledge the contribution of staff members at the Analytical Cytometry Core and DNA/RNA Synthesis Core. This work is supported by the National Cancer Institute of the National Institutes of Health under grant numbers P50CA107399 (S.F.) and P30CA033572 (City of Hope). This work is also supported through sponsored research from Scopus Biopharma, Inc. The content is solely the responsibility of the authors and does not necessarily represent the official views of the 10.13039/100000002National Institutes of Health. This study was also supported by the Billy and Audrey Wilder Endowment to H.Y. The diagrams in Figures 1E, 3C, and 5B were created with BioRender.com.

## Author contributions

H.Y. and C.Z. developed the concept, designed the experiments, and prepared and wrote the manuscript. C.Z. provided guidance and carried out the experiments and statistical analyses. R.H. contributed to animal experiments and immunofluorescence staining. L.R. performed animal experiments, real-time qPCR, and flow cytometry experiments. A.M. performed immunofluorescence staining. J.S. performed animal experiments and the real-time qPCR assay. P.S. synthesized CpG-*Stat3* siRNA. S.F. provided clinical insight on B cell lymphoma treatment, and M.K. contributed to the manuscript writing and discussion of the project.

## Declaration of interests

M.K. and H.Y. are on the scientific advisory board of Scopus Biopharma, Inc., a licensee of the CpG-*STAT3*siRNA technology, with stock options. C.Z. also received stocks through CpG-*STAT3*siRNA technology licensing. M.K. is a scientific advisor to Duet Biotherapeutics, Inc.

## Supplemental information

Document S1. Figures S1–S5

Document S2. Article plus supplemental information

## References

[1] Huang A.C., Zappasodi R. (2022). A decade of checkpoint blockade immunotherapy in melanoma: understanding the molecular basis for immune sensitivity and resistance. Nat. Immunol..

[2] Morad G., Helmink B.A., Sharma P., Wargo J.A. (2021). Hallmarks of response, resistance, and toxicity to immune checkpoint blockade. Cell.

[3] Perdikis-Prati S., Sheikh S., Bouroumeau A., Lang N. (2023). Efficacy of Immune Checkpoint Blockade and Biomarkers of Response in Lymphoma: A Narrative Review. Biomedicines.

[4] Chen P.L., Roh W., Reuben A., Cooper Z.A., Spencer C.N., Prieto P.A., Miller J.P., Bassett R.L., Gopalakrishnan V., Wani K. (2016). Analysis of Immune Signatures in Longitudinal Tumor Samples Yields Insight into Biomarkers of Response and Mechanisms of Resistance to Immune Checkpoint Blockade. Cancer Discov..

[5] Kamphorst A.O., Wieland A., Nasti T., Yang S., Zhang R., Barber D.L., Konieczny B.T., Daugherty C.Z., Koenig L., Yu K. (2017). Rescue of exhausted CD8 T cells by PD-1-targeted therapies is CD28-dependent. Science.

[6] Armengol M., Santos J.C., Fernandez-Serrano M., Profitos-Peleja N., Ribeiro M.L., Roue G. (2021). Immune-Checkpoint Inhibitors in B-Cell Lymphoma. Cancers.

[7] Lim S.Y., Shklovskaya E., Lee J.H., Pedersen B., Stewart A., Ming Z., Irvine M., Shivalingam B., Saw R.P.M., Menzies A.M. (2023). The molecular and functional landscape of resistance to immune checkpoint blockade in melanoma. Nat. Commun..

[8] Ayers M., Lunceford J., Nebozhyn M., Murphy E., Loboda A., Kaufman D.R., Albright A., Cheng J.D., Kang S.P., Shankaran V., Piha-Paul S.A. (2017). IFN-gamma-related mRNA profile predicts clinical response to PD-1 blockade. J. Clin. Invest..

[9] Grasso C.S., Tsoi J., Onyshchenko M., Abril-Rodriguez G., Ross-Macdonald P., Wind-Rotolo M., Champhekar A., Medina E., Torrejon D.Y., Shin D.S. (2020). Conserved Interferon-gamma Signaling Drives Clinical Response to Immune Checkpoint Blockade Therapy in Melanoma. Cancer Cell.

[10] Zhang C., Yue C., Herrmann A., Song J., Egelston C., Wang T., Zhang Z., Li W., Lee H., Aftabizadeh M. (2020). STAT_3_ Activation-Induced Fatty Acid Oxidation in CD8^+^ T Effector Cells Is Critical for Obesity-Promoted Breast Tumor Growth. Cell Metabol..

[11] Herrmann A., Priceman S.J., Swiderski P., Kujawski M., Xin H., Cherryholmes G.A., Zhang W., Zhang C., Lahtz C., Kowolik C. (2014). CTLA4 aptamer delivers STAT3 siRNA to tumor-associated and malignant T cells. J. Clin. Invest..

[12] Yu H., Kortylewski M., Pardoll D. (2007). Crosstalk between cancer and immune cells: role of STAT3 in the tumour microenvironment. Nat. Rev. Immunol..

[13] Kortylewski M., Kujawski M., Wang T., Wei S., Zhang S., Pilon-Thomas S., Niu G., Kay H., Mulé J., Kerr W.G. (2005). Inhibiting Stat3 signaling in the hematopoietic system elicits multicomponent antitumor immunity. Nat. Med..

[14] Kujawski M., Kortylewski M., Lee H., Herrmann A., Kay H., Yu H. (2008). Stat3 mediates myeloid cell-dependent tumor angiogenesis in mice. J. Clin. Invest..

[15] Wang T., Niu G., Kortylewski M., Burdelya L., Shain K., Zhang S., Bhattacharya R., Gabrilovich D., Heller R., Coppola D. (2004). Regulation of the innate and adaptive immune responses by Stat-3 signaling in tumor cells. Nat. Med..

[16] Yu H., Jove R. (2004). The STATs of cancer--new molecular targets come of age. Nat. Rev. Cancer.

[17] Deng J., Liu Y., Lee H., Herrmann A., Zhang W., Zhang C., Shen S., Priceman S.J., Kujawski M., Pal S.K. (2012). S1PR1-STAT3 signaling is crucial for myeloid cell colonization at future metastatic sites. Cancer Cell.

[18] Kortylewski M., Xin H., Kujawski M., Lee H., Liu Y., Harris T., Drake C., Pardoll D., Yu H. (2009). Regulation of the IL-23 and IL-12 balance by Stat3 signaling in the tumor microenvironment. Cancer Cell.

[19] Lee H., Deng J., Kujawski M., Yang C., Liu Y., Herrmann A., Kortylewski M., Horne D., Somlo G., Forman S. (2010). STAT3-induced S1PR1 expression is crucial for persistent STAT3 activation in tumors. Nat. Med..

[20] Kortylewski M., Swiderski P., Herrmann A., Wang L., Kowolik C., Kujawski M., Lee H., Scuto A., Liu Y., Yang C. (2009). In vivo delivery of siRNA to immune cells by conjugation to a TLR9 agonist enhances antitumor immune responses. Nat. Biotechnol..

[21] Lee H., Herrmann A., Deng J.H., Kujawski M., Niu G., Li Z., Forman S., Jove R., Pardoll D.M., Yu H. (2009). Persistently activated Stat3 maintains constitutive NF-kappaB activity in tumors. Cancer Cell.

[22] Priceman S.J., Kujawski M., Shen S., Cherryholmes G.A., Lee H., Zhang C., Kruper L., Mortimer J., Jove R., Riggs A.D., Yu H. (2013). Regulation of adipose tissue T cell subsets by Stat3 is crucial for diet-induced obesity and insulin resistance. Proc. Natl. Acad. Sci. USA.

[23] Zhang C., Xin H., Zhang W., Yazaki P.J., Zhang Z., Le K., Li W., Lee H., Kwak L., Forman S. (2016). CD5 Binds to Interleukin-6 and Induces a Feed-Forward Loop with the Transcription Factor STAT3 in B Cells to Promote Cancer. Immunity.

[24] Scuto A., Kujawski M., Kowolik C., Krymskaya L., Wang L., Weiss L.M., Digiusto D., Yu H., Forman S., Jove R. (2011). STAT3 inhibition is a therapeutic strategy for ABC-like diffuse large B-cell lymphoma. Cancer Res..

[25] Zhang Q., Hossain D.M., Nechaev S., Kozlowska A., Zhang W., Liu Y., Kowolik C.M., Swiderski P., Rossi J.J., Forman S. (2013). TLR9-mediated siRNA delivery for targeting of normal and malignant human hematopoietic cells *in vivo*. Blood.

[26] Zhao X., Zhang Z., Moreira D., Su Y.L., Won H., Adamus T., Dong Z., Liang Y., Yin H.H., Swiderski P. (2018). B Cell Lymphoma Immunotherapy Using TLR9-Targeted Oligonucleotide STAT3 Inhibitors. Mol. Ther..

[27] Li X., Wei Y., Wei X. (2020). Napabucasin, a novel inhibitor of STAT3, inhibits growth and synergises with doxorubicin in diffuse large B-cell lymphoma. Cancer Lett..

[28] Herrmann A., Cherryholmes G., Schroeder A., Phallen J., Alizadeh D., Xin H., Wang T., Lee H., Lahtz C., Swiderski P. (2014). TLR9 is critical for glioma stem cell maintenance and targeting. Cancer Res..

[29] Herrmann A., Kortylewski M., Kujawski M., Zhang C., Reckamp K., Armstrong B., Wang L., Kowolik C., Deng J., Figlin R., Yu H. (2010). Targeting Stat3 in the myeloid compartment drastically improves the *in vivo* antitumor functions of adoptively transferred T cells. Cancer Res..

[30] Kortylewski M., Kujawski M., Herrmann A., Yang C., Wang L., Liu Y., Salcedo R., Yu H. (2009). Toll-like receptor 9 activation of signal transducer and activator of transcription 3 constrains its agonist-based immunotherapy. Cancer Res..

[31] Herrmann A., Lahtz C., Nagao T., Song J.Y., Chan W.C., Lee H., Yue C., Look T., Mülfarth R., Li W. (2017). CTLA4 Promotes Tyk2-STAT3-Dependent B-cell Oncogenicity. Cancer Res..

[32] Garris C.S., Arlauckas S.P., Kohler R.H., Trefny M.P., Garren S., Piot C., Engblom C., Pfirschke C., Siwicki M., Gungabeesoon J. (2018). Successful Anti-PD-1 Cancer Immunotherapy Requires T Cell-Dendritic Cell Crosstalk Involving the Cytokines IFN-gamma and IL-12. Immunity.

[33] Hsu F.J., Komarovskaya M. (2002). CTLA4 blockade maximizes antitumor T-cell activation by dendritic cells presenting idiotype protein or opsonized anti-CD20 antibody-coated lymphoma cells. J. Immunother..

[34] Kepp O., Marabelle A., Zitvogel L., Kroemer G. (2020). Oncolysis without viruses - inducing systemic anticancer immune responses with local therapies. Nat. Rev. Clin. Oncol..

[35] Broomfield S.A., van der Most R.G., Prosser A.C., Mahendran S., Tovey M.G., Smyth M.J., Robinson B.W.S., Currie A.J. (2009). Locally administered TLR7 agonists drive systemic antitumor immune responses that are enhanced by anti-CD40 immunotherapy. J. Immunol..

[36] Oba T., Makino K., Kajihara R., Yokoi T., Araki R., Abe M., Minderman H., Chang A.E., Odunsi K., Ito F. (2021). In situ delivery of iPSC-derived dendritic cells with local radiotherapy generates systemic antitumor immunity and potentiates PD-L1 blockade in preclinical poorly immunogenic tumor models. J. Immunother. Cancer.

[37] Reilley M.J., Morrow B., Ager C.R., Liu A., Hong D.S., Curran M.A. (2019). TLR9 activation cooperates with T cell checkpoint blockade to regress poorly immunogenic melanoma. J. Immunother. Cancer.

[38] Gao C., Kozlowska A., Nechaev S., Li H., Zhang Q., Hossain D.M.S., Kowolik C.M., Chu P., Swiderski P., Diamond D.J. (2013). TLR9 signaling in the tumor microenvironment initiates cancer recurrence after radiotherapy. Cancer Res..

[39] Krauth J.S., Charitansky H., Isaac S., Bobin J.Y. (2006). Clinical implications of axillary sentinel lymph node 'micrometastases' in breast cancer. Eur. J. Surg. Oncol..

[40] Schmidt U., Wagner H., Miethke T. (1999). CpG-DNA upregulates the major acute-phase proteins SAA and SAP. Cell Microbiol..

[41] von Beust B.R., Johansen P., Smith K.A., Bot A., Storni T., Kündig T. (2005). Improving the therapeutic index of CpG oligodeoxynucleotides by intralymphatic administration. Eur. J. Immunol..

[42] van der Leun A.M., Thommen D.S., Schumacher T.N. (2020). CD8(+) T cell states in human cancer: insights from single-cell analysis. Nat. Rev. Cancer.

[43] Velotti F., Barchetta I., Cimini F.A., Cavallo M.G. (2020). Granzyme B in Inflammatory Diseases: Apoptosis, Inflammation, Extracellular Matrix Remodeling, Epithelial-to-Mesenchymal Transition and Fibrosis. Front. Immunol..

[44] Wensink A.C., Hack C.E., Bovenschen N. (2015). Granzymes regulate proinflammatory cytokine responses. J. Immunol..

[45] Boulch M., Cazaux M., Cuffel A., Guerin M.V., Garcia Z., Alonso R., Lemaître F., Beer A., Corre B., Menger L. (2023). Tumor-intrinsic sensitivity to the pro-apoptotic effects of IFN-gamma is a major determinant of CD_4_^+^ CAR T-cell antitumor activity. Nat. Can. (Ott.).

[46] Goodman A., Patel S.P., Kurzrock R. (2017). PD-1-PD-L1 immune-checkpoint blockade in B-cell lymphomas. Nat. Rev. Clin. Oncol..

[47] Hammerich L., Binder A., Brody J.D. (2015). In situ vaccination: Cancer immunotherapy both personalized and off-the-shelf. Mol. Oncol..

[48] Proskurina A.S., Ruzanova V.S., Ritter G.S., Efremov Y.R., Mustafin Z.S., Lashin S.A., Burakova E.A., Fokina A.A., Zatsepin T.S., Stetsenko D.A. (2022). Antitumor efficacy of multi-target *in situ* vaccinations with CpG oligodeoxynucleotides, anti-OX40, anti-PD1 antibodies, and aptamers. J. Biomed. Res..

[49] Moreira D., Adamus T., Zhao X., Su Y.L., Zhang Z., White S.V., Swiderski P., Lu X., DePinho R.A., Pal S.K., Kortylewski M. (2018). STAT3 Inhibition Combined with CpG Immunostimulation Activates Antitumor Immunity to Eradicate Genetically Distinct Castration-Resistant Prostate Cancers. Clin. Cancer Res..

[50] Sagiv-Barfi I., Czerwinski D.K., Levy S., Alam I.S., Mayer A.T., Gambhir S.S., Levy R. (2018). Eradication of spontaneous malignancy by local immunotherapy. Sci. Transl. Med..

[51] Sagiv-Barfi I., Kohrt H.E., Burckhardt L., Czerwinski D.K., Levy R. (2015). Ibrutinib enhances the antitumor immune response induced by intratumoral injection of a TLR9 ligand in mouse lymphoma. Blood.

[52] Krieg A.M. (2012). CpG still rocks! Update on an accidental drug. Nucleic Acid Therapeut..

[53] Tahir S.A., Gao J., Miura Y., Blando J., Tidwell R.S.S., Zhao H., Subudhi S.K., Tawbi H., Keung E., Wargo J. (2019). Autoimmune antibodies correlate with immune checkpoint therapy-induced toxicities. Proc. Natl. Acad. Sci. USA.

[54] Ganesh S., Kim M.J., Lee J., Feng X., Ule K., Mahan A., Krishnan H.S., Wang Z., Anzahaee M.Y., Singhal G. (2024). RNAi mediated silencing of STAT3/PD-L1 in tumor-associated immune cells induces robust anti-tumor effects in immunotherapy resistant tumors. Mol. Ther..

[55] Ehexige E., Bao M., Bazarjav P., Yu X., Xiao H., Han S., Baigude H. (2020). Silencing of STAT3 via Peptidomimetic LNP-Mediated Systemic Delivery of RNAi Downregulates PD-L1 and Inhibits Melanoma Growth. Biomolecules.

[56] Fu X.Q., Liu B., Wang Y.P., Li J.K., Zhu P.L., Li T., Tse K.W., Chou J.Y., Yin C.L., Bai J.X. (2020). Activation of STAT3 is a key event in TLR4 signaling-mediated melanoma progression. Cell Death Dis..

[57] Liu Y.X., Xu B.W., Niu X.D., Chen Y.J., Fu X.Q., Wang X.Q., Yin C.L., Chou J.Y., Li J.K., Wu J.Y. (2022). Inhibition of Src/STAT3 signaling-mediated angiogenesis is involved in the anti-melanoma effects of dioscin. Pharmacol. Res..

[58] Nechaev S., Gao C., Moreira D., Swiderski P., Jozwiak A., Kowolik C.M., Zhou J., Armstrong B., Raubitschek A., Rossi J.J., Kortylewski M. (2013). Intracellular processing of immunostimulatory CpG-siRNA: Toll-like receptor 9 facilitates siRNA dicing and endosomal escape. J. Contr. Release.

