# Supplementary material for: Local CpG-Stat3 siRNA treatment improves antitumor effects of immune checkpoint inhibitors
Source: Mol Ther Nucleic Acids. 2024 Oct 9;35(4):102357. doi: 10.1016/j.omtn.2024.102357 (PMC11605413; doi:10.1016/j.omtn.2024.102357)
Supplement: Document S1. Figures S1–S5 [file mmc1.pdf]

OMTN, Volume *35*

Supplemental information

Local CpG-*Stat3* siRNA treatment improves antitumor effects of immune checkpoint inhibitors

Chunyan Zhang, Rui Huang, Lyuzhi Ren, Antons Martincuks, JiEun Song, Marcin Kortylewski, Piotr Swiderski, Stephen J. Forman, and Hua Yu

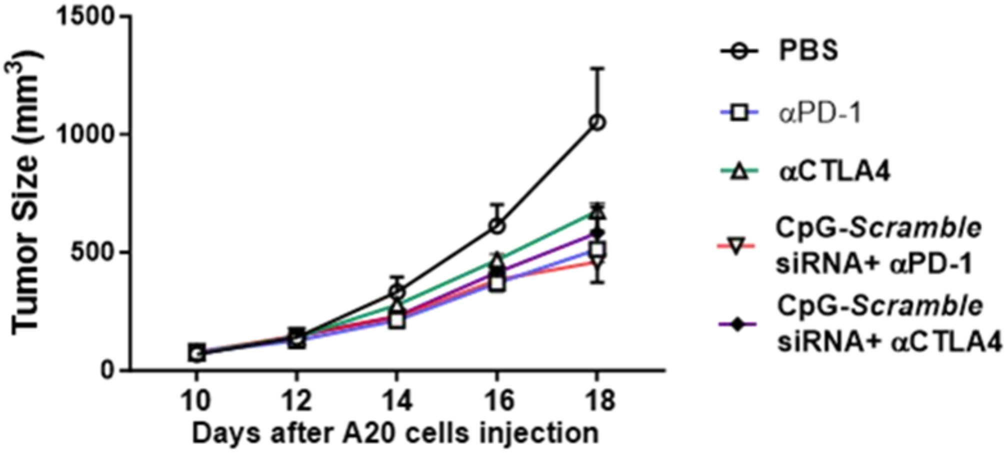

Figure S1. Anti-tumor effect of CpG-*Scramble* siRNA with or without CTLA4 or PD-1 blockade. BALB/c mice with s.c. A20 lymphoma tumors were treated by intra-tumoral injections of CpG-*Scramble* siRNA, i.p injection of anti-CTLA4, anti-PD-1 antibodies or combination treatment with CpG-*Scramble* siRNA and anti-CTLA4 or anti-PD-1 antibodies every other day, starting 10 day after tumor implantation (5 x 10^5 A20 cells/tumor). Tumor size was monitored every other day.

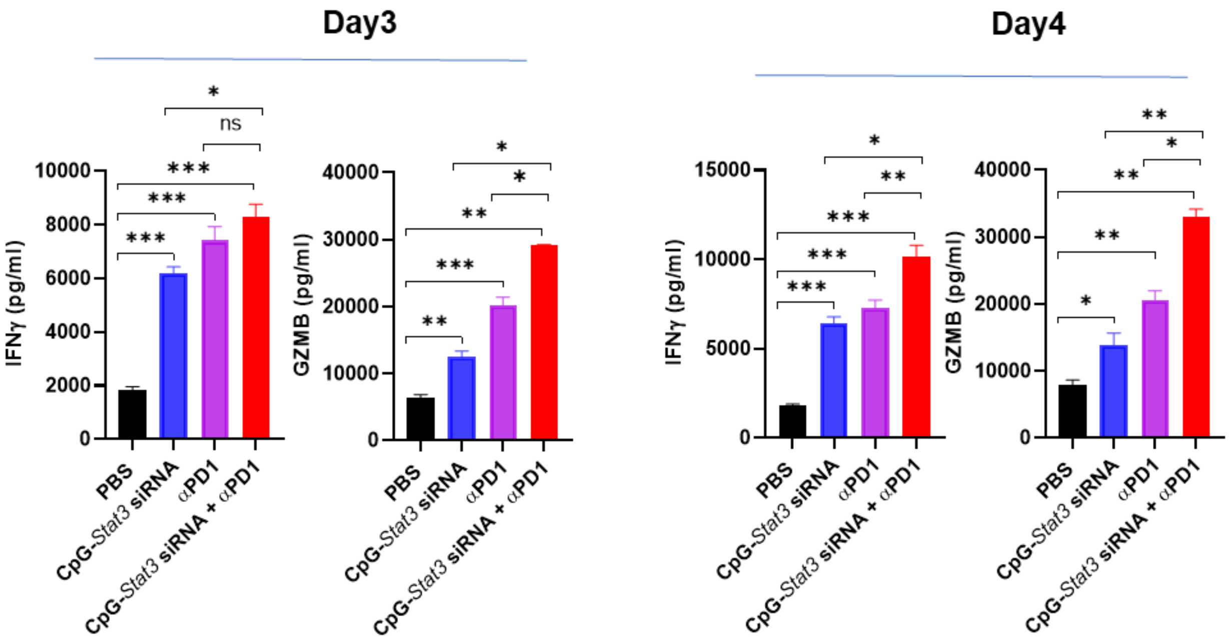

Figure S2 : Systematic IFN-γ production and GZMB expression after intra-tumoral CpG-*Stat3* siRNA and systemic PD-1 antibody single or combined treatments. Splenic cells from B16 tumor bearing mice with different treatment were co-cultured with B16 tumor cells for 3 or 4 days. The supernatants from co-culture cells were collected. ELISA assays of the co-cultured supernatant were performed to quantify secreted IFNγ and Granzyme B, both of which are the main cytotoxic molecules released by effector T cells. Data were shown with means ± SEM, n=2-3 (n is for number of samples, each of which was pooled from 2-4 mice. Student`s *t*-test was used for statistical analysis (*P<0.05; **P<0.01; ***P<0.001).

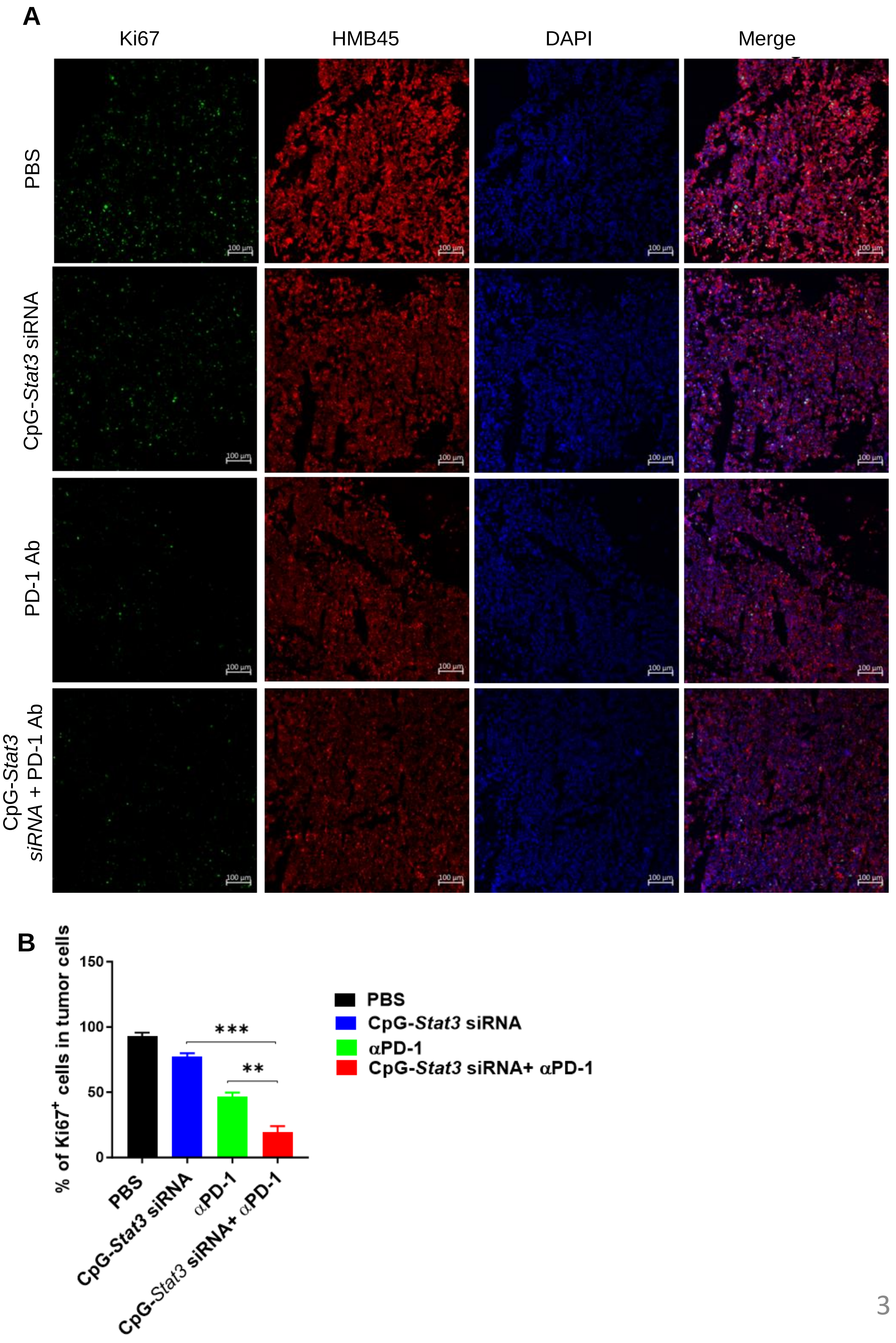

A
Ki67
HMB45
DAPI
Merge
PBS
CpG-*Stat3* siRNA
PD-1 Ab
CpG-*Stat3* *siRNA* + PD-1 Ab
100 μm
B
% of Ki67+ cells in tumor cells
150
100
50
0
***
**
PBS
CpG-*Stat3* siRNA
αPD-1
CpG-*Stat3* siRNA+ αPD-1

Figure S3 : Effects of intra-tumoral CpG-*Stat3* siRNA and systemic PD-1 antibody treatments on tumor cell proliferation. The subcutaneous human melanoma A2058 xenograft tumors (tumor cells and freshly isolated human PBMC) from NSG mice were subjected to immunofluorescent staining by anti-Ki67 and anti-HBM45 antibodies, followed by confocal microscopic analyses. A. Representative Ki67 (green) and HBM45 (red). B. Quantification of $Ki67^+$ cells in the $HMB45^+$ tumor cells. Data were shown with means ± SEM, n=3-5. N is number of tumor sections in each group (each of which was pooled from 2-3 mice). positive signals in 2-5 areas per tissue section were averaged). Student`s *t*-test was used for statistical analysis (*P<0.05; **P<0.01; ***P<0.001).

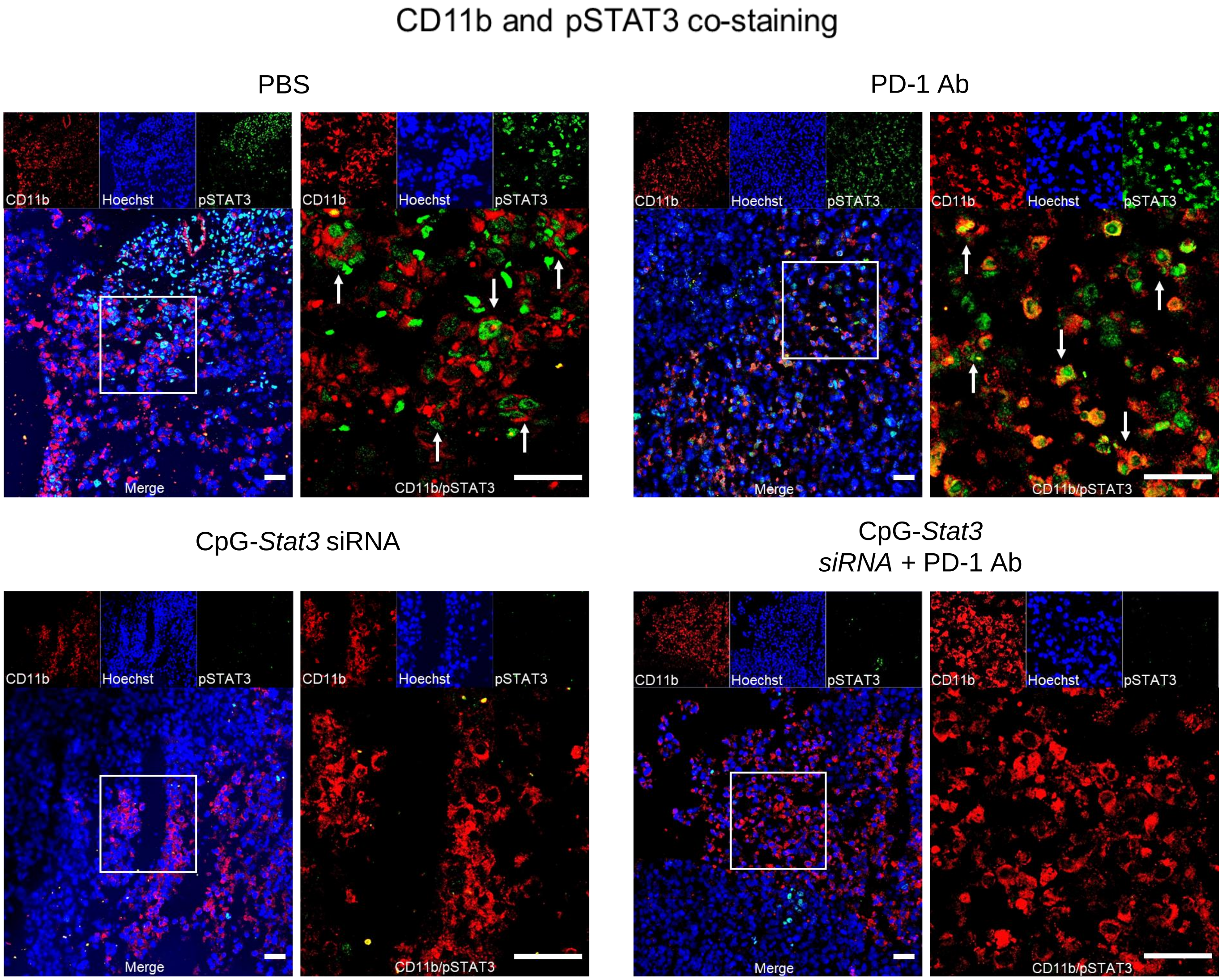

Figure S4 : pSTAT3 depletion in CD11b$^+$ myeloid cell after intra-tumoral CpG-*Stat3* siRNA treatment. Representative immunofluorescence images of CD11b (red), p-STAT3 (green) and Hoechst (blue) of tumor tissue sections from NSG mice given A2058 tumor cells and hPBMC co-transplantation. Scale bars represent 50 um.

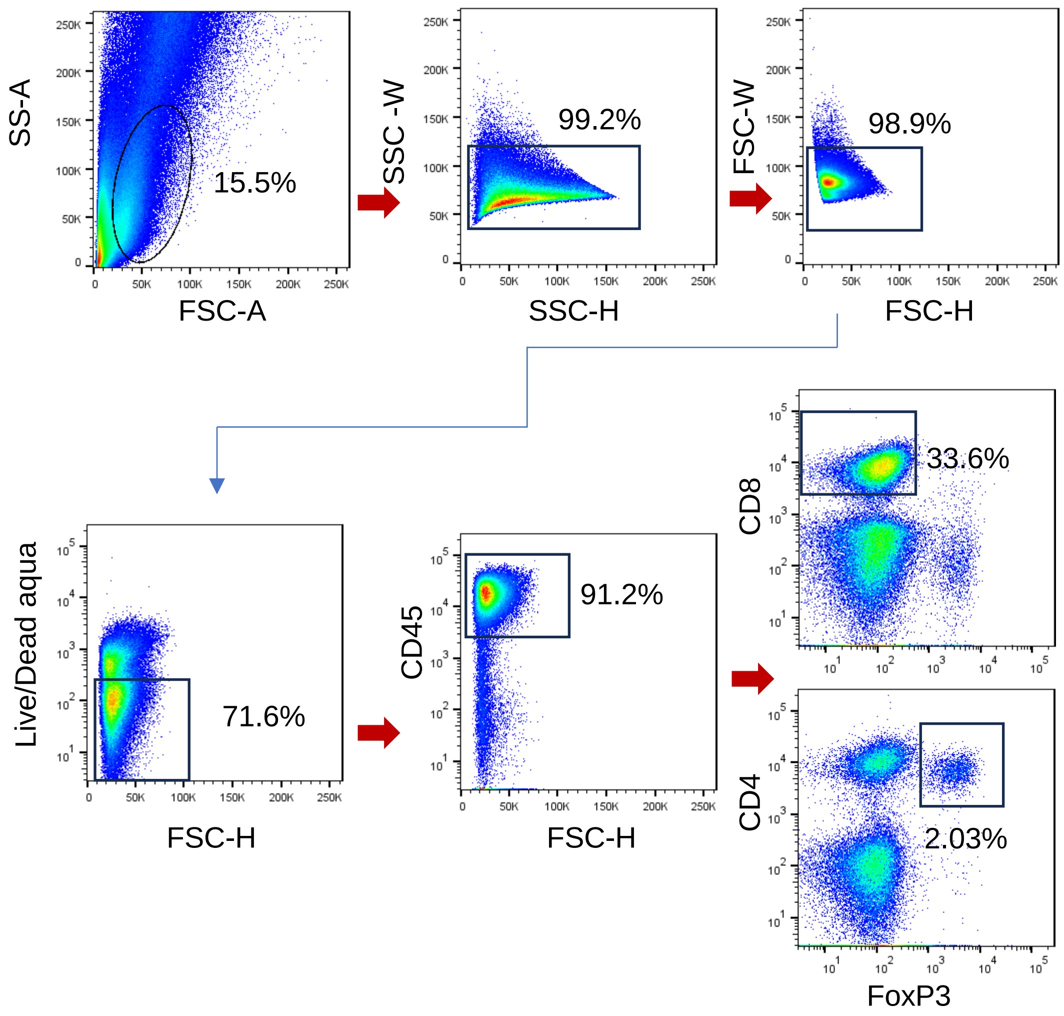

Figure S5 : Gating strategy of immune cell populations in single cell suspension from tumor tissues.
